# Collaboration processes and perceived effectiveness of integrated care projects in primary care: a longitudinal mixed-methods study

**DOI:** 10.1186/s12913-015-1125-4

**Published:** 2015-10-09

**Authors:** Pim P. Valentijn, Dirk Ruwaard, Hubertus J M Vrijhoef, Antoinette de Bont, Rosa Y. Arends, Marc A. Bruijnzeels

**Affiliations:** 1 Jan van Es Institute, Netherlands Expert Centre Integrated Primary Care, Wisselweg 33, 1314 CB Almere, The Netherlands; 2 Scientific Centre for Care and Welfare (Tranzo), Tilburg University, Tilburg, The Netherlands; 3 Department of Health Services Research, School for Public Health and Primary Care, Faculty of Health, Medicine and Life Sciences, Maastricht University, Maastricht, The Netherlands; 4 Saw Swee Hock School of Public Health, National University of Singapore, Singapore, Singapore; 5 Institute of Health Policy and Management, Erasmus University Rotterdam, Rotterdam, The Netherlands; 6 Department of Psychology, Health & Technology, University of Twente, Enschede, The Netherlands

**Keywords:** Integrated care, Primary care, Classification, Coordination of care, Collaboration, Organization models and delivery of care

## Abstract

**Background:**

Collaborative partnerships are considered an essential strategy for integrating local disjointed health and social services. Currently, little evidence is available on how integrated care arrangements between professionals and organisations are achieved through the evolution of collaboration processes over time. The first aim was to develop a typology of integrated care projects (ICPs) based on the final degree of integration as perceived by multiple stakeholders. The second aim was to study how types of integration differ in changes of collaboration processes over time and final perceived effectiveness.

**Methods:**

A longitudinal mixed-methods study design based on two data sources (surveys and interviews) was used to identify the perceived degree of integration and patterns in collaboration among 42 ICPs in primary care in The Netherlands. We used cluster analysis to identify distinct subgroups of ICPs based on the final perceived degree of integration from a professional, organisational and system perspective. With the use of ANOVAs, the subgroups were contrasted based on: 1) changes in collaboration processes over time (shared ambition, interests and mutual gains, relationship dynamics, organisational dynamics and process management) and 2) final perceived effectiveness (i.e. rated success) at the professional, organisational and system levels.

**Results:**

The ICPs were classified into three subgroups with: ‘United Integration Perspectives (UIP)’, ‘Disunited Integration Perspectives (DIP)’ and ‘Professional-oriented Integration Perspectives (PIP)’. ICPs within the UIP subgroup made the strongest increase in trust-based (mutual gains and relationship dynamics) as well as control-based (organisational dynamics and process management) collaboration processes and had the highest overall effectiveness rates. On the other hand, ICPs with the DIP subgroup decreased on collaboration processes and had the lowest overall effectiveness rates. ICPs within the PIP subgroup increased in control-based collaboration processes (organisational dynamics and process management) and had the highest effectiveness rates at the professional level.

**Conclusions:**

The differences across the three subgroups in terms of the development of collaboration processes and the final perceived effectiveness provide evidence that united stakeholders’ perspectives are achieved through a constructive collaboration process over time. Disunited perspectives at the professional, organisation and system levels can be aligned by both trust-based and control-based collaboration processes.

**Electronic supplementary material:**

The online version of this article (doi:10.1186/s12913-015-1125-4) contains supplementary material, which is available to authorized users.

## Background

Integration of health and social services is widely recognized as an essential strategy for enhancing the sustainability and affordability of any health care system [1–3]. A number of leading primary care models exist today as examples of integrated care approaches, such as the Patient-Centered Medical Homes (PCHM) and Accountable Care Organisations (ACOs) in the United States, Primary Care Trusts (PCT) in the English National Health Service (NHS), and Community Health Centres and Care Groups in The Netherlands [4–7]. Primary care is considered the cornerstone upon which various health and social services can be built [1, 8], and it has proven to be essential for achieving desired health outcomes and limiting costs [3, 9]. Throughout this paper, we refer to *integrated primary care* as ambulatory care settings in which multiple professionals and organisations across the health and social care system provide accessible, comprehensive and coordinated services to a population in a community [10]. Despite the increasing popularity of integrated care, there is a lack of knowledge on how integrated care can effectively be implemented in a primary care setting [8, 11, 12].

Early academic research on integrated care has mainly focused on centralised top-down implementation strategies (e.g. regulatory frameworks, contractual mechanisms), which failed to demonstrate improved outcomes and highlighted the difficulties of aligning various actors (e.g. policymakers, managers, organisations, professionals) across multiple settings [13–16]. More recently scholars argue that bottom-up collaborative approaches (e.g. partnerships and networks) might be more effective strategies to implement integrated care [11, 16–21].

The underlying assumption is that effective implementation strategies are linked to relational ‘trust-based’ (e.g. trust, mutual respect and shared values), rather than to functional ‘control-based’ (e.g. formal rules and structures) integration mechanisms [18, 22]. Within a primary care context, trust-based collaboration approaches from the bottom-up are considered essential for stimulating the integration of different services [8, 10, 11] because they have traditionally been delivered by professionally-owned, disjointed, small-scale practices [23].

However, empirical evidence regarding the collaboration processes that underlie the development of integrated care in a primary care setting is scarce [10]. Within integrated care studies, the collaboration process towards integrated care is often evaluated as a “black box,” with little understanding of the critical mechanisms for success or failure [11, 24]. This knowledge gap makes it difficult to understand and explain how the evolution of collaboration processes serves as a means to develop integrated care, thus restraining the opportunities to identify effective implementation strategies. This knowledge is of utmost importance, as implementing integrated care through collaborative partnerships is described as time-consuming, resource intensive, and fraught with challenges [19, 21, 25]. Consequently, there is a need to identify the underlying collaboration processes over time to better understand how integrated care can effectively be implemented among and between different professional and organisational groups.

In this study, we draw from the Rainbow Model of Integrated Care (RMIC) [8] to define the concept of integrated care and use the model of Bell, Kaats and Opheij [26] to describe the collaboration processes over time. The RMIC defines integrated care from four different perspectives and levels: 1) *clinical or service integration* (patient or client perspective at the micro level), 2) *professional integration* (professional perspective at the meso level), 3) *organisational integration* (managerial perspective at the meso level), and 4) *system integration* (policymaker perspective and policy climate at the macro level). The four key domains provide a framework to characterise the degree of integration from a multifocal perspective. Within the present study, the RMIC is used to explore possible differences among integration perspectives between the stakeholders’ at the clinical, professional, organisational and system levels. The literature suggests that a similarity of integration perspectives by multiple stakeholders is needed in order for an integrated care initiative to be effectively implemented [16, 27]. Similarly, analysing the underlying changes in collaboration processes helps to understand the way in which (dis)similarities of integration perspectives between stakeholders are achieved [10, 11].

The conceptual model of Bell et al. [26] explicates five dimensions for evaluating a collaboration process: 1) *Shared ambition* (shared commitment), 2) *Mutual gains* (acknowledgement of the various interests), 3) *Relationship dynamics* (relational capital), 4) *Organisation dynamics* (shared control), and 5) *Process management* (process steering). The model is developed within the field of inter-organisational management science and is grounded on a solid base of literature in which the different dimensions have been described [28–32]. In a previous study, we found that these five collaboration process dimensions were a powerful way to predict the final perceived effectiveness (i.e. rated success) of integrated care projects (ICPs) in The Netherlands [10].

The first aim of this study was to develop an exploratory typology of ICPs based on the perceived degree of integration of stakeholders at the professional, organisational and system levels. The second aim was to study how the types of integration differ in changes of collaboration processes over time and final perceived effectiveness. The following research questions were addressed: *1) Which subgroups of ICPs can be distinguished based on the final perceived degree of integration from a professional, organisational and system perspective?; 2) To what extent do these subgroups of integration differ with regard to changes in the five collaboration processes over time?; and 3) To what extent do these subgroups of integration differ with regard to the perceived effectiveness (*i.e. *rated success) of an ICP from the professional, managerial and policy perspective?*

## Methods

### Study design and setting

The present study was a longitudinal mixed-methods study conducted among ICPs enrolled in the Dutch national integrated primary care programme *Op één lijn* (translated as “Primary Focus”) [33]. The Primary Focus programme aimed to stimulate integration through collaboration among community health and social services in a primary care setting by funding 69 ICPs across The Netherlands. Selected ICPs received a service grant in 2010 (*n* = 50) or in 2011 (*n* = 19) by the Netherlands Organization for Health Research and Development (ZonMw). A full description of the selection criteria can be found in a previous publication [10]. As part of the programme, the ICPs participated in a longitudinal study (from 2010 to 2013) that aimed to assess the changes in collaboration processes as well as the integration arrangements that were foreseen to arise at the end of the programme. The average funding period of the participating 69 ICPs was 22.9 months (SD 7.5, range 5–36) and the average time between programme measurement point at the start (T0) and end (T1) was 19.5 months (SD 7.3, range: 6–38).

To be eligible for the present study, ICPs had to meet the following two criteria: 1) consist of a form of inter/intra-sectorial, inter/intra-organisational and/or inter/intra-professional collaboration among different professionals (such as general practitioners (GP), nurses and social workers) and/or organisations (such as GP practices, nursing homes, long-term care facilities, and hospitals) and 2) have data available regarding the degree of integration as perceived by professionals, the steering committee and the external evaluators (interviewers). Based on the selection criteria, 42 out of 69 ICPs were selected (61 %).

Although initially planned it was not feasible to include the patient perspective, as due to various reasons (e.g. resistance among ICPs, questionnaire inappropriate for specific population) data was only available for 26 out of the 69 ICPs (38 %). As a result, the patient perspective was excluded from further analysis in the present study.

### Data collection procedure

Both surveys and interviews were used to examine the changes in collaboration processes, the final perceived degree of integration and the effectiveness of the ICPs. At the system level every project coordinator was included and two additional stakeholders per ICP (using purposive sampling), and at the organisational and professional levels all participants of an ICP were included. Table 1 provides a summary of the data collection procedure.

**Table 1:** Overview data collection procedure

Levels	Participants	Measurement methods	Data processing	Variables (Time points)
System	Project coordinator and two stakeholders	Semi-structured interviews	Coding by use of code book^a^	System integration (T1)
				Perceived effectiveness (T1)
Organisational	Steering committee members	Questionnaire	Aggregated means at ICP level	Organisational integration (T1)
				Perceived effectiveness (T1)
				Collaboration process (T0 - T1)
Professional	Frontline professionals	Questionnaire	Aggregated means at ICP level	Professional integration (T1)
				Perceived effectiveness (T1)

^a^For details of the coding process see Additional file 1

#### System level

The degree of system integration and the perceived effectiveness from a policy perspective was evaluated using semi-structured interviews held by process evaluators at the end (T1) of the funding period of each ICP. Eight process evaluators conducted semi-structured interviews with the project coordinator and two stakeholders per ICP. The stakeholders were selected based on their central position in the implementation process of an ICP as identified by the project coordinator. The interviews were transcribed and coded by the same process evaluator who conducted the interviews using a step-by-step thematic analysis procedure to enable an overall quantitative analysis. An interview scheme was used to obtain information about the fit between the strategic objectives and the policy conditions (e.g. public laws and regulations) and the final success of the ICP (see Additional file 1 for the interview scheme). Data was transcribed in a priori developed qualitative template using excel processing software (see Additional file 1 for an example of a qualitative template). Qualitative data was coded using the coding structure derived from the process evaluation interviews conducted at the start of the program. The interview data were merged per ICP by the process evaluator, who also provided written comments and interpretations of exemplar quotes per participant and recurring themes across participants. A structured case report for each ICP was written consisting of a narrative summary of all information obtained. The participants were emailed a copy of the case report to review and verify for accuracy. Subsequently, the process evaluator rated the degree of system integration and the final success of the ICP on a standardised coding scheme using the content from the case reports.

#### Organisational level

The degree of organisational integration, the changes in collaboration processes, and the perceived effectiveness from a managerial point of view were examined using questionnaires completed by the steering committee members of each ICP. Steering committee members were defined as partners who were involved in the administration and strategic decision making processes of the ICP as identified in the original grant application, and verified by the project coordinator.

To explore the changes in the collaboration process dimensions, a questionnaire was sent by e-mail at the start (T0) and end (T1) of the funding period of each ICP. The questionnaire at T0 contained questions about the collaboration process dimensions, and the modified questionnaire at T1 consisted also of questions regarding information about the final perceived degree of integration effectiveness of the ICP (Details of the questionnaire are provided in the next section “Measures.”). An e-mail reminder was sent to non-respondents after 1 week.

#### Professional level

The degree of professional integration and the perceived effectiveness from a professional point of view was measured using questionnaires completed by frontline professionals at T1 of the funding period of each ICP. Questionnaires were sent by e-mail to all active frontline professionals of an ICP as identified by the project coordinator. A frontline professional was defined as any health or social professional (such as GP, nurse, social worker and allied health professionals) who took part in the frontline service delivery of the ICP. Reminders were sent to non-respondents after 2 weeks and again after 4 weeks.

### Measures

#### System level

A standardised coding scheme was used to analyse the degree of system integration and the success of the ICP from a policy perspective. The external process evaluators (interviewers) identified the degree of system integration from the interviews, which was defined as the perceived degree to which the implementation of an ICP was facilitated by public laws and regulations. Interviewers coded: (1) the extent to which public laws and regulations facilitated the implementation of the ICP on a 3-point scale ranging from one (not at all) to three (completely), and (2) the overall perceived effectiveness of the ICP on a scale ranging from one (unsuccessful) to five (very successful) based on the content of the template of each ICP. Details about the three steps and standardized documents used to quantitatively code the degree of system integration and the perceived effectiveness can be found in Additional file 1.

#### Organisational level

A questionnaire based on the model of Bell et al. [26] that was developed and validated by the authors in a previous study [10] was used to measure the five collaboration process dimensions at T0 and T1. For each collaboration dimension (shared ambition, mutual gains, relationship dynamics, organisational dynamics and process management), steering committee members indicated the extent to which he/she agreed with a given statement on a 4-point Likert-scale ranging from 1 (not at all) to 4 (totally). Details about the individual items of the collaboration process variables can be found in an additional publication [10]. Changes in collaboration processes were calculated as follows: (score on T1 minus score on T0)/ score on T0.

The questionnaire sent at T1 was complemented with items to measure the perceived degree of organisational integration. Organisational integration was defined as “the extent to which the steering committee members experience a collectively elaborated inter-organisational strategy within the ICP.” Organisational integration was assessed with a four item, 4-point Likert scale ranging from one (not at all) to four (totally). Details of the individual items of the organisational integration variable are provided in Table 1. The internal consistency and reliability of the organisation integration scale was tested during the current study. Finally, steering committee members were asked to rate the overall perceived effectiveness of the ICP on a scale from zero (unsuccessful) to ten (very successful).

#### Professional level

Professional integration was defined as “the extent to which frontline professionals experienced a shared agreement on interdisciplinary service delivery.” Items for measuring professional integration were taken from existing surveys that address coordinated service delivery in a primary care setting [34, 35]. Response categories ranged on a 5-point Likert scale from one (very unsatisfied) to five (very satisfied). Details of the items used to measure professional integration are listed in Table 1. The internal consistency and reliability of the professional integration scale was analysed during the current study. Finally, professionals were asked to rate the overall perceived effectiveness of the ICP on a scale from one (unsuccessful) to five (very successful).

The aggregated means, minimum, maximum and standard deviations of above mentioned measures can be found in Table 2.

**Table 2:** Characteristics of the variables

				Baseline (T0)		Follow-up (T1)		Change (T1-T0/T0)	
Variable	No. of items	Range (lowest score- highest score)	Cronbach’s Alpha	Mean	SD	Mean	SD	Mean	SD
Degree of integration									
System integration^a^	1	1–3	NA	NA	NA	2.70	0.39	NA	NA
Organisational integration^b^	4	1–4	0.82	NA	NA	3.31	0.33	NA	NA
Professional integration^c^	10	1–5	0.88	NA	NA	3.52	0.27	NA	NA
Change in collaboration process^d^									
∆ Shared ambition	4	1–4	0.78	3.50	0.25	3.48	0.26	0.00	0.09
∆ Mutual gains	4	1–5	0.82	3.06	0.34	3.01	0.40	−0.01	0.14
∆ Relationship dynamics	4	1–6	0.71	3.22	0.29	3.27	0.35	0.02	0.12
∆ Organisation dynamics	6	1–7	0.86	3.05	0.29	3.09	0.35	0.02	0.14
∆ Process management	4	1–8	0.82	3.05	0.29	3.12	0.35	0.03	0.13
Success of the project									
Perceived success at system level	1	1–5	NA	NA	NA	3,67	0,79	NA	NA
Perceived success at organisational level	1	0–10	NA	NA	NA	7.22	0.90	NA	NA
Perceived success at professional level	1	1–5	NA	NA	NA	4.05	0.36	NA	NA

*NA* not assessed

^a^An acceptable level of inter-rater reliability (κ = .60) was found for the system integration variable

^b^Items: a) Is the aim of the inter-organisational arrangement explicated? b) Are the solution(s) of the inter-organisational arrangement explicated? c) Are the chances of the inter-organisational arrangement explicated? and d) Is the form for the inter-organisational arrangement explicated? Single-factor solution, with factor loadings ranging from: 0.798 to 0.832

^c^Items: a) Leadership is being demonstrated. b) Are roles and tasks of the team clear? c) Are final clinical goals agreed upon? d) Do information systems (ICT) support the team’s functionality? e) Are there agreements about the interdisciplinary care delivery? f) Are outcomes for the patients clear? g) Are outcomes for the professionals clear? h) Are outcomes for the community clear. i) Is the interdisciplinary approach applicable elsewhere. j) Is the effectiveness of the inter-professional team clear? Single-factor solution, with factor loadings ranging from: 0.348 to 0.784

^d^Details about the items of the collaboration process variables can be found in an additional publication [10]

### Data analyses

A randomly selected sample of five ICPs (12 %) was additionally coded by an author (PV) to explore the inter-rater reliability for the system integration variable. A Cohen’s κ of .60 was found between the codes from the author and the interviewers, indicating an acceptable level of inter-rater reliability [36]. For the organisational integration, professional integration and collaboration process scales (shared ambition, mutual gains, relationship dynamics, organisational dynamics and process management), a maximum of 30 % missing answers per scale at the individual response level was tolerated [10]. Then, the validity and reliability of the organisational and professional integration variables was tested at the individual response level. To analyse the internal consistency, an exploratory principal-component factor analysis with varimax rotation was conducted, using a threshold of ≥ .30 to identify items that clustered together (see Table 1, Notes). Cronbach’s alphas ranged from .71 for the relationship dynamic scale and .88 for the professional integration scale.

Subsequently, individual responses were aggregated to the project level as the ICP level was the primary unit of analysis. To determine whether data collected from the individual level could be aggregated to represent the views at the ICP level, the within-partnership variance was examined in relation to the between-partnership variance by using a one-way analysis of variance (ANOVA) [19, 37]. Project-level scores were obtained by calculating the scale score for each respondent and then taking the average score across all of the respondents within a project [19]. The within-partnership variance was significantly less (*p* ≤ .01) in relation to the between-partnership variance for the professional integration, organisational integration, collaboration process and effectiveness variables. These findings indicate that the mean scores could be aggregated to the ICP level [19, 37].

A cluster analysis was conducted using the system, organisational and professional integration variables. Pearson correlation was assessed to check for multicollinearity between the variables (all correlations were <0.5) [38, 39]. As each variable was measured on a different scale, standardization was necessary prior to each variable entering into the cluster analyses [40]. A three-step procedure was followed for clustering the ICPs in different subgroups [40, 41]. First, the appropriate number of clusters was determined by means of hierarchical cluster analysis using Ward’s method and the Euclidean Distance. An increase in the agglomeration coefficient indicated a large jump in within-cluster variability, providing strong support for a three-cluster solution. Second, a non-hierarchical analysis was performed (K-means method) to validate and adjust the results of the hierarchical procedures, using the initial cluster centroids from Ward’s method as seed points [40, 42–44]. Third, the stability of the cluster assignment between the Ward’s and the K-means method was assessed using Cohen’s κ coefficient of agreement [36, 41]. Results indicated a perfect agreement (κ = 1.00, *p* < .001), suggesting both methods produced a similar cluster solution of the ICPs [41]. The cluster means for each of the three integration variables were used to provide a meaningful interpretation of the clusters [40].

To assess differences in the degree of integration, change in collaboration processes and perceived effectiveness among the three subgroups (clusters), multivariate and univariate analyses of variance with Bonferroni-adjusted post-hoc comparisons were used. Given the exploratory nature of this study, *p*-values between .05 and .10 were considered suggestive for an association, whereas *p*-values < .05 were considered statistically significant. All data analyses were performed using the Statistical Package for the Social Sciences version 21 for Windows (IBM Statistics).

### Ethics

The study design of the Primary Focus programme was reviewed by the Independent Review Board Nijmegen (IRBN) [45]. The committee concluded that further ethical approval was not needed according to the Dutch Medical Research in Human Subjects Act (WMO). There were no ethical objections raised against the study. All participants were asked permission verbally or in writing to participate in the study.

## Results

### Sample characteristics

Table 3 shows the general characteristics of the included ICPs. Among the 42 ICPs, a total of 126 interviews were conducted at the system level. The overall individual response at the organisational level was 71 % (235 out of 330 questionnaires) at T0, and 78 % (229 out of 294 questionnaires) at T1. At the professional level, the overall individual response was 37 % (468 out of 1279 questionnaires) at T1.

**Table 3:** General characteristics of the 42 ICPs

Funding configurations	
Funding period by agency (months), mean (SD), range	22.31 (7.31), 5–36
Funding by agency (€), mean (SD), range	89.154 (36.622), 32.930–188.892
Scope and objectives	
Geographic scope, *n* (%)	
Local community level	33 (78.6)
Regional province level	9 (21.4)
Objective, *n* (%)	
Chronic care	10 (23.8)
Elderly	7 (16.7)
Local collaboration	12 (28.6)
Integrating health and social services	7 (16.7)
Other	6 (14.2)
Organisational configuration	
Prior history of collaboration, *n* (%)	
Yes	38 (90.5)
No	4 (9.5)
Own investment, *n* (%)	
Yes	8 (19.0)
No	34 (81.0)
Legally formalised, *n* (%)	
Yes	7 (16.7)
No	35 (83.3)

### Profiling the perceived degree of integration (research question 1)

To answer the first research question, three distinctive subgroups of integration were identified across the ICPs. Table 4 presents the mean scores for the integration variables per subgroup. Results of the between-subgroup post-hoc comparisons identified statistically significant differences between the subgroups for the perceived degree of system integration (*F*(2, 39) = 26.67, *p <* .001), organisational integration (*F*(2, 39) = 21.58, *p* < .001) and professional integration (*F*(2, 39) = 9.18, *p <* .01). The three subgroups were named according to their average characteristics regarding the perceived degree of integration.

**Table 4:** Characteristics of the subgroups and tests of differences between groups

	Total	Subgroup 1 United Integration Perspectives (UIP)	Subgroup 2 Disunited Integration Perspectives (DIP)	Subgroup 3 Professional-oriented Integration Perspectives (PIP)	Subgroup differences
*n* (%)	42	16 (38.1)	9 (21.4)	17 (40.5)	F-tests
Degree of integration - mean (SD)					Multivariate F (6, 76) = 25.95^***^
System integration	2.69 (0.38)	3.00 (0.00)a	2.89 (0.22)a	2.29 (0.25)b	F (2, 39) = 26.67^***^
Organisational integration	3.29 (0.34)	3.52 (0.17)a	2.87 (0.22)b	3.30 (0.29)c	F (2, 39) = 21.58^***^
Professional integration	3.52 (0.28)	3.61 (0.19)a	3.23 (0.26)b	3.60 (0.28)a	F (2, 39) = 9.18^**^
Change in collaboration process - mean (SD)					Multivariate F (10, 72) = 1.57
Shared ambition	0.01 (0.10)	0.02 (0.06)	−0.03 (0.10)	0.02 (0.10)	F(2, 39) = 0.96
Mutual gains	−0.00 (0.12)	0.04 (0.09)a	−0.10 (0.11)b	0.01 (0.12)	F(2, 39) = 4.44^*^
Relationship dynamics	0.03 (0.10)	0.05 (0.07)a	−0.05 (0.12)b	0.04 (0.10)	F(2, 39) = 3.82^*^
Organisation dynamics	0.03 (0.12)	0.06 (0.09)a	−0.08 (0.12)b	0.05 (0.11)a	F(2, 39) = 5.42^**^
Process management	0.03 (0.12)	0.04 (0.09)	−0.07 (0.14)a	0.08 (0.10)b	F(2, 39) = 5.68^**^
Perceived effectiveness - mean (SD)					Multivariate F (6, 70) = 4.93^***^
System level	3.76 (0.66)	4.07 (0.46)a	3.22 (0.97)b	3.76 (0.44)	F(2, 38) = 5.63^**^
Organisational level	7.20 (0.84)	7.72 (0.37)a	6.2 (0.93)b	7.23 (0.66)a	F(2, 39) = 16.43^***^
Professional level	4.01 (0.33)	4.02 (0.28)	3.71 (0.18)a	4.15 (0.36)b	F(2, 36) = 5.70^**^

^*^ = *p* < .05; ^**^ = *p* < .01; ^***^ = *p* < .001. Means that do not share the same subscript (a, b or c) differ in the Bonferroni-adjusted post-hoc comparisons (*p* < .05)

### Subgroup 1: United Integration Perspectives (UIP)

ICPs in this group comprised 31.8 % (*n* = 16) of the sample. They were characterized by system, organisational and professional integration scores above average (see Table 3), and thus labelled as “United Integration Perspectives (UIP).” ICPs in this subgroup exhibited significantly higher scores on the organisation integration perspective (*M* = 3.52, *SD* = 0.17) than ICPs in subgroup 2 and subgroup 3 (*M* = 2.87, *SD* = 0.22; *M* = 3.30, *SD* = 0.29 respectively).

### Subgroup 2: Disunited Integration Perspectives (DIP)

ICPs in this subgroup comprised 21.4 % (*n* = 9) of the sample and were characterized by average scores on system and professional integration combined with relatively low organisational integration scores (see Table 4). This subgroup was interpreted as “Disunited Integration Perspectives (DIP).” ICPs in subgroup 2 were characterized by significantly lower levels on the organisation integration perspective (*M* = 2.87, *SD* = 0.22) compared to subgroup 1 (*M* = 3.52, *SD* = 0.17) and subgroup 3 (*M* = 3.30, *SD* = 0.29). Moreover, subgroup 2 also exhibited significantly lower scores on the professional integration perspective (*M* = 3.23, *SD* = 0.26) than ICPs in subgroup 1 and subgroup 3 (*M* = 3.61, *SD* = 0.19; *M* = 3.60, *SD* = 0.28 respectively).

### Subgroup 3: Professional-oriented Integration Perspectives (PIP)

ICPs in this group comprised 40.5 % (*n* = 17) of the sample. ICPs were characterized by low system integration scores, average organisation integration scores and high professional integration scores (see Table 4), and thus labelled as “Professional-oriented Integration Perspective (PIP).” ICPs in subgroup 3 exhibited significant lower scores on the system integration perspective (*M* = 2.29, *SD* = 0.25) than ICPs in subgroup 1 and subgroup 2 (*M* = 3.00, *SD* = 0.00; *M* = 2.89, *SD* = 0.22 respectively).

### Change in collaboration processes (research question 2)

No significant differences were found between subgroups in change in collaboration process variables with the multivariate test (*F*(10,72) = 1.57, ns). Significant differences were found between subgroups for changes over time in mutual gains (*F*(2, 39) = 4.44, *p* = .02); relationship dynamics (*F*(2, 39) = 3.82, *p* = .03); organisation dynamics (*F*(2, 39) = 5.42, *p* = .008) and process management (*F*(2, 39) = 5.68, *p* = .007). No statistically significant differences between the subgroups were found in regard to change in shared ambitions (*F*(2, 39) = 0.96, ns).

### Subgroup 1: United Integration Perspectives (UIP)

ICPs in subgroup 1 made a substantial increase in the collaboration process variables over time (see Table 4). Post hoc comparisons revealed that ICPs in subgroup 1 exhibited a significant increase in mutual gains (*M* = 0.04, *SD* = 0.09) and relationship dynamics (*M* = 0.05, *SD* = 0.07) over time compared to ICPs in subgroup 2 (*M* = −0.10, *SD* = 0.11; *M* = −0.05, *SD* = 0.12, respectively). The increases in shared ambition and process management over time did not significant differ compared to ICPs in subgroup 2 and 3.

### Subgroup 2: Disunited Integration Perspectives (DIP)

ICPs in subgroup 2 were characterized by a substantial decrease in the collaboration process variables over time (see Table 4). Interestingly, ICPs in subgroup 2 exhibited a significant decrease in organisational dynamics (M = −0.08, SD = 0.12) compared to ICPs in subgroup 1 (M = 0.06, SD = 0.09) and subgroup 3 (M = 0.05, SD = 0.11). ICPs in subgroup 2 also showed a significantly decrease in mutual gains (*M* = −0.10, *SD* = 0.11) and relationship dynamics (*M* = −0.05, *SD* = 0.12) over time compared to the ICPs in subgroup 1 (*M* = 0.04, *SD* = 0.09; *M* = 0.05, *SD* = 0.07, respectively). Moreover, subgroup 2 ICPs also exhibited a significant decrease in process management (*M* = −0.07, *SD* = 0.14) over time compared to ICPs in subgroup 3 (*M* = 0.08, *SD* = 0.10).

### Subgroup 3: Professional-oriented Integration Perspectives (PIP)

The collaboration process variables in subgroup 3 increased over time (see Table 4). ICPs in subgroup 3 exhibited significantly higher scores on organisational dynamics (*M* = 0.05, *SD* = 0.11) then ICPs in subgroup 2 (*M* = −0.08, *SD* = 0.12), but did not differ in shared ambition, mutual gains and relationship dynamics scores compared to subgroup 1 and 2 (see Table 3). However, ICPs in subgroup 3 had significant higher scores on process management (*M* = 0.08, *SD* = 0.10) over time compared to the ICPs in subgroup 2 (*M* = −0.07, *SD* = 0.14).

### Perceived effectiveness (research question 3)

The subgroups differed significant on perceived effectiveness among ICPs using a multivariate test *(F* (6, 70) = 4.93, *p <* .001). Significant differences between subgroups were found for the perceived effectiveness at system (*F*(2, 38) = 5.63, *p* = .007), organisational (*F*(2, 39) = 16.43, *p <* .001) and professional (*F*(2, 36) = 5.70, *p* = .007) levels.

### Subgroup 1: United Integration Perspectives (UIP)

ICPs in subgroup 1 were characterized by average effectiveness rates at the professional level combined with high effectiveness rates at the organisational and system level (see Table 3). Post hoc comparisons indicated that ICPs in subgroup 1 exhibited significant higher effectiveness rates at the organisational level (*M* = 7.72, *SD* = 0.37) and system level (*M* = 4.07, *SD* = 0.46) compared with ICPs in subgroup 2 (*M* = 6.20, *SD* = 0.93; *M* = 3.22, *SD* = 0.97, respectively).

### Subgroup 2: Disunited Integration Perspectives (DIP)

ICPs in subgroup 2 were characterized by relatively low effectiveness scores at the system, organisational and professional level (see Table 4). ICPs in subgroup 2 exhibited significant lower effectiveness rates at the system level (*M* = 3.22, *SD* = 0.97) compared to ICPs in subgroup 1 (*M* = 4.07, *SD* = 0.46). Moreover, they also exhibited the lowest organisational effectiveness rates (*M* = 6.20, *SD* = 0.93) compared to ICP’s in subgroup 1 (*M* = 7.72, *SD* = 0.37) and subgroup 3 (*M* = 7.23, *SD* = 0.66). Finally, they also exhibited significant lower effectiveness scores at the professional level (*M* = 3.71, *SD* = 0.18) compared to ICPs in subgroup 3 (*M* = 4.15, *SD* = 0.36).

### Subgroup 3: Professional-oriented Integration Perspectives (PIP)

ICPs in subgroup 3 were characterized by average effectiveness scores at the system and organisational level combined with high effectiveness rates at the professional level (see Table 3). ICPs in subgroup 3 exhibited significant higher effectiveness rates at the organisational (*M* = 7.23, *SD* = 0.66) and professional (*M* = 4.15, *SD* = 0.36) level compared to ICP’s in subgroup 2 (*M* = 6.20, *SD* = 0.93; *M* = 3.71, *SD* = 0.18, respectively).

## Discussion

Based on the perceived degree of integration from a multiple stakeholders’ perspective (professionals, managers and policymakers), ICPs were segmented into three subgroups, which we named: ‘United Integration Perspectives (UIP)’, ‘Disunited Integration Perspectives (DIP)’ and ‘Professional-oriented Integration Perspectives (PIP)’. The ICPs within the *UIP subgroup* were perceived as most effective, had the highest perceived degree of integration at the organisational and system levels and average scores at the professional level. The *DIP subgroup* ICPs were characterized with the lowest perceived effectiveness, lowest degree of integration at all levels. The ICPs within the *PIP subgroup* were characterised by an average degree of perceived effectiveness, lowest perceived degree of integration at the system level and average scores at the organisational and professional level. Both the *UIP* and *PIP subgroups* showed an increase in collaboration processes over time. ICPs within the *DIP subgroup* were characterized by a decrease in collaboration processes over time.

### Contribution of research findings

These findings support the recent theories form the literature that the effectiveness of an ICP is improved when all stakeholders (professionals, managers and policymakers) are aligned. In other words, our study highlights the need to develop a multilayer commitment from professionals, organisations and system actors when leading integrated care efforts [2, 8, 11, 16, 22, 27, 46, 47].

Furthermore, ICPs within the *PIP subgroup* showed a gap between professional and system perspectives in the development of integrated care. The ICP interviewees’ low perceived degree of system integration and relatively high degree of professional integration as well as the high perceived effectiveness at the professional level displays different integration perspectives of professional and system level stakeholders. The literature suggests that environmental policy conditions (e.g. public laws and regulations) can be counteracting forces in achieving operational integration goals [1, 16, 48]. In this context, the low degree of system integration may indicate that local health policy reforms (e.g. transitions from the Exceptional Medical Expenses Act to the existing Social Support Act and new Long-Term Care Act in The Netherlands) [49] during the ‘Primary Focus programme’ may have had a negative effect upon ICPs in the *PIP subgroup.* However, much variation existed between the objectives of the ICPs and the complexity of their system environment (e.g. urban vs. rural). Future studies should focus in more detail on how system features interact with the content of integration initiatives.

Another explanation for the opposing system integration and professional integration effectiveness scores could be that there is a glass ceiling at the organisational level when developing integrated care in practice. The observed changes in the collaboration processes over time as well as the degree of organisational integration between the *PIP* and *UIP subgroups* indicated that the development towards integrated care varied. For example, ICPs within the *PIP subgroup* displayed a strong increase in ‘transactional’ control-based (organisational dynamics and process management) collaboration mechanisms over time. Arguably, the organisational level (steering committees) of these ICPs were focused on controlling power struggles and/or conflicts of interests particularly associated with developing integrated care across professional boundaries [11, 16–21, 50]. In comparison, ICPs within the *UIP subgroup* showed a strong increase in ‘relational’ trust-based (mutual gains and relationship dynamics) collaboration approaches in addition to the control-based mechanisms. Interestingly, the *UIP subgroup* showed the strongest increase in the mutual gains approach, suggesting that the effectiveness of an ICP improves when the conflicting interests and motives across professionals, managers and policymakers are successfully aligned and bridged at the organisational level [10, 16, 26, 29]. This might suggest that both relational trust- and functional control-based collaboration processes are of crucial importance to successfully develop and align integration efforts at the professional, organisational and system levels.

Finally, no significant differences in shared ambition (e.g. vision and mission of the ICPs) between the three subgroups were found, even though a shared consensus on the collaboration purpose is considered an essential process condition for achieving integrated care [16, 27, 51]. One reason that we did not found any differences between the shared ambition among the subgroups might be related to the selection criteria of the funding agency, in that, in order to be selected, the majority of projects had to show a prior history of collaboration [10]. Consistent with our previous study, this finding indicates that a shared ambition is rather a precondition then a crucial process condition of an effective integration strategy, since integrated care initiatives all begin as a shared vision by all the stakeholders [10].

### Strengths and weaknesses of this study

Identifying subgroups of ICPs using cluster analysis has provided an excellent way to study the complex nature of integrated care through a multifocal perspective. Likewise, incorporating three different actor-group perspectives holds more external validity and generalizability than studying integrated care from one perspective only (as conducted in earlier studies). The present study provides persuasive empirical evidence for a typology of integrated care and how integrated care is effectively developed through changes in collaboration processes over time. The differences across the three subgroups in terms of the development of their collaboration processes and their final perceived effectiveness provides valuable implementation knowledge in the burgeoning field of integrated primary care.

Several limitations of the present study are notable. This study highlighted the problems associated with collecting data from multiple stakeholders’ perspectives. As noted earlier, patient experiences of integrated care could, unfortunately, not be included, mostly due to resistance among ICPs to measure the degree of integration among their patients. Although the selection criteria of the funding agency explicitly stated that patients’ should be central in the integration process, the majority of the ICPs argued that measuring the experience of care was not part of the Primary Focus programme because the principle objectives were focused on governance structures [10]. Including the patient perspective is important not only for its positive association with patient safety and clinical effectiveness [52], but also as an organising principle to restructure services around the needs and values of people [1, 8]. Since patients tend to have different preferences compared to other stakeholder groups [53], selection bias is likely to have influenced the construction of the ICPs typology. Further validation of the link between the typology of ICPs and the patient experience of integrated care is, therefore, required. Only a limited number of studies have attempted to describe or evaluate the concept of integrated care from the perspective of patients [54], in part, by a lack of research methods. This study must therefore be seen as an important first step towards future multilevel evaluation studies that incorporate the patient perspective in addition to the professional, managerial and policy perspectives on integrated care.

Due to the principal objectives of the Primary Focus programme, the research team established better relationships at the organisational level compared to the professional and clinical level. This is reflected in the higher response rate at organisational level (87 %) compared to the professional level (37 %). The organisational level, and hence the managerial perspective might, be overrepresented in the present study. We are aware of the fact that the possible existence of selection and response bias could have influenced the results of our study. For example, we were only able to measure the development of collaboration processes at the organisational level. As a consequence, positive results regarding the collaboration process might be overrepresented by the steering committee members in order to be perceived more favourably for future funding [10]. Caution should, therefore, be taken when generalising the results of this study, because effective integration strategies in one setting may not be transferable to other settings (e.g. secondary and tertiary care) and countries, due to differing cultural and organisational contexts [2, 55].

Finally, this study assessed the various actors’ perceptions about their ICP behaviours; whether these behaviours actually do affect the outcomes, and in what fashion, remains to be empirically tested. The present study used the stakeholders’ effectiveness perspectives as a proxy for the ICP performance. By definition, the use of self-reported data involves risks of social desirability and differences in recall [10, 56]. Further research is needed to link the typology of ICPs with more objective health and cost-related outcomes. Unfortunately, this particular analysis could not be done in the present study because the necessary data was unavailable. Nevertheless, the academic literature has only just begun to understand and study the complex field of integrated primary care through a multifocal perspective and the results derived thus far encourage further research.

### Implications for practice and future research

This study provides valuable implementation knowledge for professionals, managers, commissioners, and policymakers on how to develop effective integration strategies in a primary care context. The typology of ICPs is an important step to understand the concept of integrated primary care and to compare different types of collaborating professionals and organisations. The typology can be used as a framework for assessing performance in terms of quality, cost and health outcomes and diagnosing the integration and collaboration characteristics across multiple types of organisations [50]. Moreover, the typology provides a potential diagnostic tool for professionals, managers, commissioners, and policymakers to analyse their integrated care arrangements’ which, subsequently, can be used to customise integrated care strategies to local circumstances to make them more effective.

The subgroups of integration found in this study emphasize the need and value of theorizing, studying and developing integrated care through a multifocal perspective. The empirical recognition that aligned stakeholders’ perspectives towards integrated care are related to changes in underlying collaboration processes supports the hypothesis that integrated care is a complex interdisciplinary, nonlinear and dynamic change process [17, 18, 22, 57]. Thus, to understand how integrated care functions, it is necessary to use and develop research methodologies that acknowledge a complex philosophy of science [58].

Future research in this area should, therefore, focus on the entire complexity of inter-relationships among all the actors involved within an integrated care initiative. In this regard, future studies should investigate in more detail the balance between opposing professional and system perspectives of integration and the need of relational trust- versus transactional control-based collaboration mechanisms to bridge the different, and sometimes polar, perspectives. Social Network Analysis (SNA) [59, 60] can be an useful aid to further study and understand these complex dynamics of integrated care. Once researchers are able to quantify and visualise these complex interactions, an understanding of which integrated care strategies can lead to better patient outcomes relative to the amount of money spent within a specific context might emerge. In their entirety, the results of this exploratory study highlight the need for cross-level theories and performance evaluations to determine how best to accelerate the progress of value-based integrated care.

## Conclusions

The typology of ICPs provides evidence that final effectiveness is improved when all stakeholders (professionals, managers and policymakers) perceive a high degree of integration. This finding highlights the need to develop a multilayer commitment when leading integrated care efforts. In this regard, both trust- and control-based collaboration processes are critical for bridging the gap of opposing integration perspectives between stakeholders at the professional and organisational system levels. Our findings underscore the value of theorizing, evaluating and developing integrated care through a multifocal perspective to enhance a more complete understanding of the best way to establish successful integrated care interventions.

## Additional file

Additional file 1: **Data collection procedure system level.** A step-by-step thematic analysis procedure was followed to enable an overall quantitative analysis for the system level variables: system integration and perceived effectiveness. The coding process was conducted in three steps using the following materials: 1) Semi-structured interview scheme, 2) Qualitative template, and 3) Coding scheme. (DOCX 17 kb)

## Abbreviations

RMIC: Rainbow model of integrated care
ICPs: Integrated care projects
GP: General practitioner
IRBN: Independent Review Board Nijmegen
WMO: Dutch Medical Research in Human Subjects Act
UIP: United integration perspectives
DIP: Disunited integration perspectives
PIP: Professional-oriented Integration Perspective
