# Supplementary material for: Collaboration processes and perceived effectiveness of integrated care projects in primary care: a longitudinal mixed-methods study
Source: BMC Health Serv Res. 2015 Oct 9;15:463. doi: 10.1186/s12913-015-1125-4 (PMC4598962; doi:10.1186/s12913-015-1125-4)
Supplement: Additional file 1: — Data collection procedure system level. A step-by-step thematic analysis procedure was followed to enable an overall quantitative analysis for the system level variables: system integration and perceived effectiveness. The coding process was conducted in three steps using the following materials: 1) Semi-structured interview scheme, 2) Qualitative template, and 3) Coding scheme. (DOCX 17 kb) [file 12913_2015_1125_MOESM1_ESM.docx]

**Additional files**

**Additional file 1 – Data collection procedure system level**

A step-by-step thematic analysis procedure was followed to enable an overall quantitative analysis for the system level variables: system integration and perceived effectiveness. The coding process was conducted in three steps using the following materials: 1) Semi-structured interview scheme, 2) Qualitative template, and 3) Coding scheme.

**Step 1: Semi-structured interview scheme**

The following interview scheme was used to obtain information about the fit between the strategic objectives and the policy conditions (e.g. public laws and regulations) and the final success of the ICPs.

**External environment of the project**

1. To what extent are policy regulations (e.g. laws and funding schemes) a barrier for achieving the goals of the project?
   - No barrier
   - Very small barrier
   - Small barrier
   - Reasonable barrier
   - Major barrier
   - I don’t know
2. Could you give an example of how policy regulations (e.g. laws and funding schemes) hamper the project?
3. Is there a solution to this barrier within your project?
4. Are there other policy regulations barring the achievement of the project goals?

**Results of the project**

1. Which results have been achieved within the project?
2. Which are you most proud of?
3. Could you give examples of the specific products and/or services that have been realised?

**Step 2: Qualitative template**

The qualitative template was used to analyse, code and summarise the interview data for each ICP (see Step 1).

**External environment of the project**

| **Are policy regulations (e.g. laws and funding schemes) a barrier for achieving the goals of the project?** | |
| --- | --- |
| **Quantitative summary** | \| **Respondent 1** \| **Respondent 2** \| **Respondent 3** \| \| --- \| --- \| --- \| \| No barrier/ Very small barrier/ Small barrier/ Reasonable barrier /Major barrier/ I don’t know \| No barrier/ Very small barrier/ Small barrier/ Reasonable barrier /Major barrier/ I don’t know \| No barrier/ Very small barrier/ Small barrier/ Reasonable barrier /Major barrier/ I don’t know \| \| **Explanatory note** \| **Explanatory note** \| **Explanatory note** \| \|  \|  \|  \| |
| **Qualitative summary** |  |

| **Results of the project** | |
| --- | --- |
| **Qualitative summary** |  |

**Step 3: Coding scheme for interviewers**

The coding scheme was used to quantitatively rate the degree of system integration and final success of the ICP using the content qualitative templates (see Step 2).

**External environment of the project**

1. **Is the implementation of the project facilitated by policy regulations (e.g. laws and funding schemes)?**

- Not at all (e.g. Public regulations hamper the implementation.)
- Partially (e.g. Public regulations partially facilitate the implementation.)
- Completely (e.g. Public regulations facilitate the implementation.)

**Results of the project**

1. **To what extent do you think the project is successfully executed (e.g. goal achievement)?**

- Very unsuccessful
- Unsuccessful
- Neutral
- Successful
- Very successful
